# A cross-species co-functional gene network underlying leaf senescence

**DOI:** 10.1093/hr/uhac251

**Published:** 2022-11-10

**Authors:** Moyang Liu, Chaocheng Guo, Kexuan Xie, Kai Chen, Jiahao Chen, Yudong Wang, Xu Wang

**Affiliations:** Shanghai Collaborative Innovation Center of Agri-Seeds/School of Agriculture and Biology, Shanghai Jiao Tong University, Shanghai 200240, China; Joint Center for Single Cell Biology, School of Agriculture and Biology, Shanghai Jiao Tong University, Shanghai 200240, China; Shanghai Collaborative Innovation Center of Agri-Seeds/School of Agriculture and Biology, Shanghai Jiao Tong University, Shanghai 200240, China; Joint Center for Single Cell Biology, School of Agriculture and Biology, Shanghai Jiao Tong University, Shanghai 200240, China; Shanghai Collaborative Innovation Center of Agri-Seeds/School of Agriculture and Biology, Shanghai Jiao Tong University, Shanghai 200240, China; Joint Center for Single Cell Biology, School of Agriculture and Biology, Shanghai Jiao Tong University, Shanghai 200240, China; Shanghai Collaborative Innovation Center of Agri-Seeds/School of Agriculture and Biology, Shanghai Jiao Tong University, Shanghai 200240, China; Joint Center for Single Cell Biology, School of Agriculture and Biology, Shanghai Jiao Tong University, Shanghai 200240, China; Shanghai Collaborative Innovation Center of Agri-Seeds/School of Agriculture and Biology, Shanghai Jiao Tong University, Shanghai 200240, China; Joint Center for Single Cell Biology, School of Agriculture and Biology, Shanghai Jiao Tong University, Shanghai 200240, China; Shanghai Collaborative Innovation Center of Agri-Seeds/School of Agriculture and Biology, Shanghai Jiao Tong University, Shanghai 200240, China; Joint Center for Single Cell Biology, School of Agriculture and Biology, Shanghai Jiao Tong University, Shanghai 200240, China; Shanghai Collaborative Innovation Center of Agri-Seeds/School of Agriculture and Biology, Shanghai Jiao Tong University, Shanghai 200240, China; Joint Center for Single Cell Biology, School of Agriculture and Biology, Shanghai Jiao Tong University, Shanghai 200240, China

## Abstract

The complex leaf senescence process is governed by various levels of transcriptional and translational regulation. Several features of the leaf senescence process are similar across species, yet the extent to which the molecular mechanisms underlying the process of leaf senescence are conserved remains unclear. Currently used experimental approaches permit the identification of individual pathways that regulate various physiological and biochemical processes; however, the large-scale regulatory network underpinning intricate processes like leaf senescence cannot be built using these methods. Here, we discovered a series of conserved genes involved in leaf senescence in a common horticultural crop (*Solanum lycopersicum*), a monocot plant (*Oryza sativa*), and a eudicot plant (*Arabidopsis thaliana*) through analyses of the evolutionary relationships and expression patterns among genes. Our analyses revealed that the genetic basis of leaf senescence is largely conserved across species. We also created a multi-omics workflow using data from more than 10 000 samples from 85 projects and constructed a leaf senescence-associated co-functional gene network with 2769 conserved, high-confidence functions. Furthermore, we found that the mitochondrial unfolded protein response (UPR^mt^) is the central biological process underlying leaf senescence. Specifically, UPR^mt^ responds to leaf senescence by maintaining mitostasis through a few cross-species conserved transcription factors (e.g. *NAC13*) and metabolites (e.g. ornithine). The co-functional network built in our study indicates that UPR^mt^ figures prominently in cross-species conserved mechanisms. Generally, the results of our study provide new insights that will aid future studies of leaf senescence.

## Introduction

Leaf senescence takes advantage of the orderly cellular process of apoptosis, and is vitally important for nutrient recycling, environmental adaptation, and reproduction [1, 2]. Diverse plant groups, including angiosperms, gymnosperms, bryophytes, green algae, and photosynthetic prokaryotes, employ senescence-like processes [3], and there is substantial variation in cell structure, metabolism, and gene expression between taxa [4]. Diverse environmental stressors can trigger leaf senescence, including nutrient deficiencies, darkness, drought, ozone, low light levels, extreme temperatures, and pathogens [5]. During the leaf senescence process, alterations in cytology and biochemistry take place, such as chloroplast disintegration as well as decreases in cytoplastic volume and cellular metabolic activity [6]. The mitochondria maintain homeostasis, mediate the breakdown of macromolecules, and redistribute resources from senescent tissue to young, proliferative, and storage tissues [4, 7, 8]. Mitostasis is maintained by the mitochondrial protein control system (mtPQC), of which the plant mitochondrial unfolded protein response (UPR^mt^) is one of the most important pathways. Transient oxidative bursts activate MAPK and hormone signals, which combine to restore mitochondrial proteostasis via the AP2, MYB, and NAC effectors [9]. Characterization of the complex processes underlying senescence is important for developing approaches to manage the potentially deleterious effects of senescence.

The process of leaf senescence is regulated at the transcriptional and translational levels, as well as pre- and post-transcription and translation [10]. Large-scale transcriptomic reprogramming is a particularly important step in the leaf senescence process [2]. In general, leaf senescence transcriptional regulation is carried out by various master transcription factors (TFs) [11, 12]. The roles of only a few TF families (e.g. *NAC* or *WRKY*) in leaf senescence have been examined in previous studies. However, investigations of the shared features of the regulatory mechanism underlying leaf senescence across species have not been widely reported [2, 13].

Tools such as COXPRESdb [14], WeGET [15], GeneMANIA [16], WGCNA [17], and CLIC [18] have been developed to utilize co-expression networks and gene expression correlations to assign putative functions to genes. One of the shortcomings of these tools is that they require correlated expression thresholds to discretely categorize genes [19], substantially impacting subsequent interpretations of the results. An alternative approach, used by the GeneBridge toolbox, is to use a broad transcriptomic dataset to assign gene functions. Specifically, the co-expression relationships between all genes and target genes are used to identify connections between modules and genes, and target gene expression across a population is treated as a continuous variable [20]. Compared with approaches utilizing correlation coefficients to infer gene functions, such as WeGET [15] and COXPRESdb [14], the GeneBridge toolbox exhibits superior predictive performance. A recent application of GeneBridge led to the discovery that the enzyme d-dopachrome tautomerase (DDT) participates in the process of mitochondrial respiration, a finding that has been confirmed experimentally through RNA interference-mediated *DDT* knockdown [20].

The degree of similarity in the central biological hub mediating leaf senescence among species remains unclear. Addressing such a question requires an integrated, multi-omics analysis across various species during several stages of development, as the senescence process is highly complex. Here, we used a combination of evolutionary, molecular biological, and computation analysis to generate a conserved co-functional leaf senescence gene network across species. Our network sheds light on how leaf senescence interacts with other biological processes. Specifically, we identified several genes involved in leaf senescence with new functions and found that mitostasis underlies the process of leaf senescence and acts as a central biological hub. Overall, the findings of our study provide new information that will aid future studies of leaf senescence.

## Results

### Functional conservation of the pathways underlying leaf senescence among species

**Figure 1:**
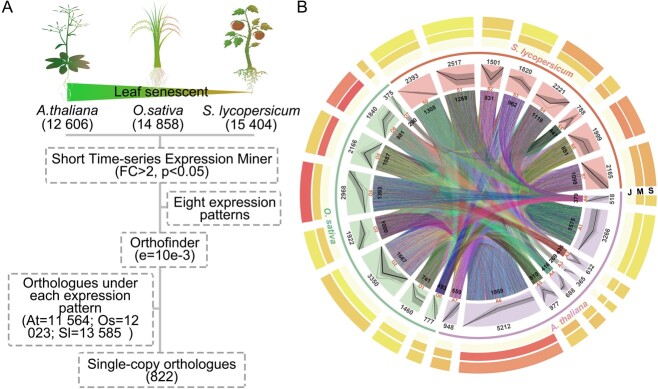
Functionally conserved leaf senescence-associated pathways. (A) Illustration of the methods used to identify conserved, cross-species, leaf senescence-associated genes. We obtained publicly available gene expression data for *S. lycopersicum*, *O. sativa*, and *A. thaliana*. The data were homogenized and the Short Time-series Expression Miner was used to quantify relative gene expression levels at the leaf maturity and senescence stages relative to the juvenile stage (*P* < .05, FC > 2). Gene clustering according to their levels of expression revealed eight EPs (EP0–EP7). The evolutionary relationships between EPs across species were evaluated using Orthofinder (e = 10e−3), resulting in the identification of 274 single-copy orthogroups. (B) EP and evolutionary relationship data were integrated to identify conserved, cross-species, leaf senescence-associated genes. Shown from outside to inside are the average values of gene expression at different developmental stages, EPs, and orthologous relationships.

The expression patterns (EPs) of genes involved in leaf senescence at different developmental stages were analyzed (Fig. 1A), as were also their evolutionary relationships (Fig. 1B). We obtained publicly available data on gene expression during three leaf development stages (juvenile, mature, and senescence) [21–23]. A total of 15 404, 12 606, and 14 858 genes were detected from *Solanum lycopersicum*, *Arabidopsis thaliana*, and *Oryza sativa*, respectively. The STEM program [24] was used to calculate the expression multiple for all periods relative to the juvenile period, which was used as a control, and the log_2_ fold change (FC) of the expression multiple was taken. We theoretically observed a total of eight EPs (3^(*n*−1)^−1) for the three developmental periods according to significantly expressed gene clustering: EP0–EP7 (Supplementary Data Table S1). Gene expression decreased during leaf senescence in EP0, EP1, and EP3. Gene expression increased during leaf senescence in EP4, EP6, and EP7. Gene expression levels were low during maturity in EP2, and high during maturity in EP5. In subsequent analyses, we focused on EP3 and EP4 because the genes in these EPs were significantly differentially expressed only in the senescence stage (Supplementary Data Fig. S1A). The percentage of genes involved in the whole developmental process exceeded 70% in all three plant species, while the percentage of these genes expressed during the senescence stage was <4% (Supplementary Data Table S1).

We used OrthoFinder to analyze the evolutionary relationships between all expressed genes in *S. lycopersicum*, *A. thaliana*, and *O. sativa* [25]. We obtained orthogroups and orthologs, constructed an orthogroup root gene tree, and identified all occurrences of gene duplication. A high percentage of genes in each species exhibited cross-species orthologs: 88% (13 585) in *S. lycopersicum*, 81% (12 023) in *O. sativa*, and 92% (11 564) in *A. thaliana* (Supplementary Data Table S2). The constructed ortholog networks varied in complexity, but overall consisted of 4570 distinct sub-networks containing many-to-many, many-to-one, one-to-many, and one-to-one orthogroups (Fig. 1B, Supplementary Data Table S3).

Gene EPs and evolutionary relationships were used to identify the EP-associated orthologs of *S. lycopersicum*, *O. sativa*, and *A. thaliana* (Supplementary Data Table S3). For example, at EP0, 169 genes in *O. sativa* were orthologous to 220 genes in *S. lycopersicum*, 36 of 518 genes in *A. thaliana* were orthologous to 43 genes in *O. sativa* (777 genes), 215 genes were orthologous to 286 genes in *S. lycopersicum* EP0 (2393 genes), and 12 genes exhibited single-copy orthogroups. In general, genes associated with any particular EP tended to be orthologous, although a few genes across all three species were found to belong to single-copy orthogroups. Overall, we discovered 274 single-copy orthogroups expressed solely during leaf senescence, and these were marked as conserved, leaf senescence-associated cross-species genes (Supplementary Data Table S4).

A Gene Ontology (GO) analysis was carried out to identify the conserved, cross-species biological processes involved in leaf senescence, using single-copy orthogroup genes from *A. thaliana* (Supplementary Data Fig. S1B, Supplementary Data Table S5). Genes associated with eight EPs were found to be enriched in several processes, including oxidation–reduction process, response to chemical, and cellular response to chemical stimulus. The oxidation–reduction process occurs in the mitochondria to provide energy for cellular processes and plays an essential role in maintaining basic life activities [26]. We also found that conserved, cross-species genes related to leaf senescence were significantly enriched in several functions, including cell communication, response to hormone, and hormone-mediated signaling. These results are consistent with studies of leaf senescence in other plant species [6, 7, 27, 28] (Supplementary Data Fig. S1C and D, Supplementary Data Table S6). Overall, we found that single-copy orthogroups across EPs and their senescence-associated biological processes and functions are functionally conserved across species.

### Leaf senescence-associated gene co-functional network

**Figure 2:**
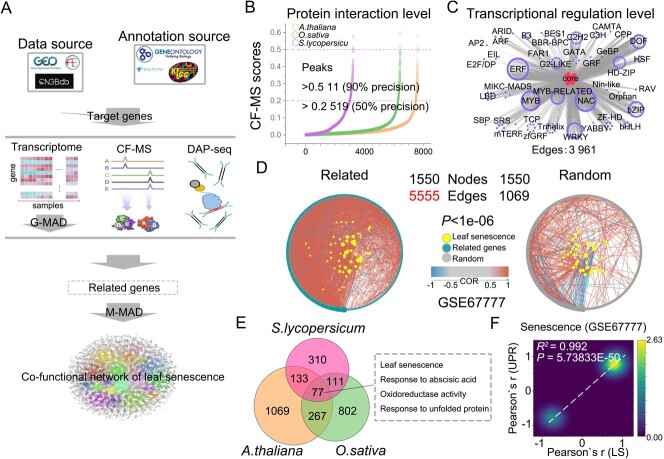
Leaf senescence-associated gene co-functional network. (A) Diagram of the workflow architecture. Public databases were used to collect multi-omics data from various species, and G-MAD, CF–MS, and DAP-seq were used to collect the genes linked to transcription, protein, and DNA–protein levels for each target. M-MAD was used to determine target gene functions. (B) CF–MS data were used to identify leaf senescence-associated genes. (C) DAP-seq data were used to identify leaf senescence-associated TFs. (D) Correlation network of related and unrelated (random) genes associated with leaf senescence (GSE67777). Statistical significance was determined by comparing correlation degree (color of edges) and network complexity (number of edges) (*P* < 1e−06). The numbers of input genes are represented by nodes. (E) Venn diagram of cross-species conserved functions related to leaf senescence in *S. lycopersicum*, *O. sativa*, and *A. thaliana*, according to M-MAD. (F) The senescence dataset (GSE67777) was used to create a correlation density map comparing leaf senescence- and unfolded protein response-associated genes. Rows indicate correlations among co-functional network genes and leaf senescence-associated genes. Columns indicate correlations among co-functional network genes and unfolded protein response-associated genes. During the process of leaf senescence, the leaf senescence- and unfolded protein response-associated genes exhibit strong interactions throughout the co-functional network.

In order to conduct a global co-functional leaf senescence-associated gene network analysis, we obtained nearly 10 000 samples of raw data from more than 85 projects from the public CNGBdb, ArrayExpress, and GEO repositories (Supplementary Data Table S7). We would like to note that the objectives and methods of these studies differed, including the use of different levels of transcription, transcriptional regulation, and protein–protein interactions. To examine associations among 274 conserved genes involved in leaf senescence at the DNA–protein, protein, and transcript levels, we used DNA affinity purification sequencing (DAP-seq) [29], co-fractionation mass spectrometry (CF–MS) [30], and Gene–Module Association Determination (G-MAD) [20] (Fig. 2A). CF–MS is a high-throughput protein interaction detection method (Fig. 2B, Supplementary Data Table S8) and DAP-seq is a high-throughput *in vitro* assay for the discovery of TF binding sites (Fig. 2C, Supplementary Data Table S9). A total of 1497 related genes were identified, including 62 known leaf senescence genes, such as *NAP* [31–33], *NAC017* [34], and *CHI* [35, 36], which demonstrates that the GeneBridge toolbox can effectively recover information about gene function (Supplementary Data Table S10).

We used correlation network analysis to further examine the public senescence dataset (GSE67777) and confirm whether expression of the related genes was correlated with the leaf senescence network (Fig. 2D, Supplementary Data Table S11). We used Module–Module Association Determination (M-MAD) [20] to determine the GO functions of the conserved, cross-species genes involved in leaf senescence and obtain module–module association scores (MMASs). Across the co-functional network, genes related to leaf senescence had 2769 functions, and 77 of these functions were shared between *A. thaliana*, *O. sativa*, and *S. lycopersicum*, including leaf senescence, macromolecular substance metabolism [6, 7, 27, 28], and several new functions, such as the response to unfolded proteins [9, 37] (Fig. 2E, Supplementary Data Table S10). Using the newly discovered association between leaf senescence and response to unfolded proteins as an example, we conducted a correlation network analysis of existing common senescence datasets (GSE67777) and found that cross-species conserved genes involved in leaf senescence also carry out functions relating to the unfolded protein response (Fig. 2F, Supplementary Data Table S11). Overall, we identified leaf senescence-associated cross-species co-functional gene networks using data on transcriptional regulation and interactions, and protein interactions.

### Systemic mitochondrial stress response is induced by leaf senescence

**Figure 3:**
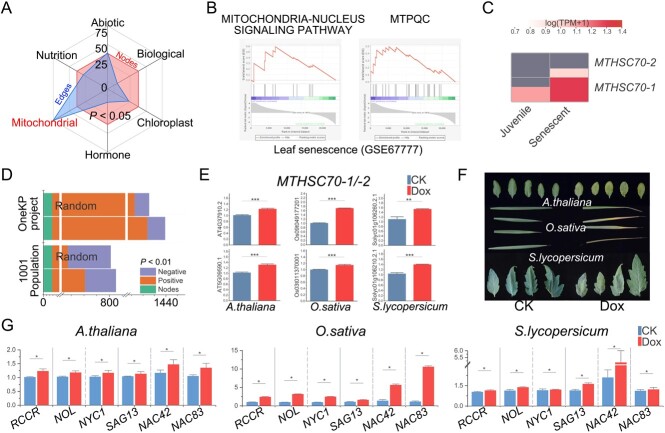
Mitochondrial stress response is induced by leaf senescence. (A) Leaf senescence-associated co-functional network genes were subjected to a microenvironment analysis (*P* < .05). (B) According to gene set enrichment analysis (GSEA) the senescence dataset (GSE67777) was enriched in gene sets involved in mitochondria–nucleus signaling and mtPQC. (C) *MTHSC70-1*/*-2*, which are marker genes of UPR^mt^, were significantly expressed during senescence. The shade of color indicates the gene expression level, and redder colors correspond to higher expression. (D) Correlation network analysis of the leaf-senescence associated co-functional gene network according to the *A. thaliana* 1KP Project and 1001 Genomes. The numbers of input genes are represented by nodes. (E) Analysis of *MTHSC70-1*/*-2* expression by qRT–PCR in *S. lycopersicum*, *O. sativa*, and *A. thaliana* under mitochondrial stress (10 mg ml^−1^ doxycycline). Data are shown as mean ± standard deviation, *n* = 3 (*P < .05). (F) Phenotypes of *S. lycopersicum*, *O. sativa*, and *A. thaliana* leaves under mitochondrial stress. (G) Expression analysis of the leaf senescence-associated genes *RCCR*, *NOL*, *NYC1*, *SAG13*, *ANAC042*, and *ANAC083* by qRT–PCR in *S. lycopersicum*, *O. sativa*, and *A. thaliana* under mitochondrial stress (10 mg ml^−1^ doxycycline). Data are shown as mean ± standard deviation, *n* = 3 (*P < .05).

Senescence involves diverse and complementary mechanisms that respond to an array of selective pressures [3]. Some of the processes involved in leaf senescence might also be involved in responses to stress, such as when plant cells experience abiotic stress, biotic stress, chloroplast stress, hormonal stress, mitochondrial stress, or nutrient stress. The leaf senescence-associated co-functional gene network was found to be closely associated with the mitochondrial stress-associated co-functional gene network (Fig. 3A, Supplementary Data Table S12), suggesting a potential link between leaf senescence and mitostasis. Gene set enrichment analysis (GSEA) showed that the mitochondria–nucleus signaling-associated, mtPQC (Fig. 3B), and UPR^mt9^ genes (*MTHSC70-1*/*-2*, which mediate the folding of mitochondrial proteins and can act as marker genes for UPR^mt^) were significantly upregulated during leaf senescence (Fig. 3C). Homeostasis and biological functioning are maintained through facilitation by these mitochondria-related pathways [9], which suggests that leaf senescence induces a systemic mitochondrial stress response.

We conducted a correlation network analysis of the leaf senescence-associated gene expression levels using the 1001 Genomes Project [38], in order to determine whether these senescence-associated genes were conserved across *A. thaliana* populations. We found that, across *A. thaliana* populations, gene expression levels were similar, and that this correlation was absent from a set of random genes (Fig. 3D and Supplementary Data Table S13). Furthermore, the 1KP program [39] was used to identify leaf senescence-associated orthologs (e = 1e−5) across major plant lineages and at least 1 billion years of evolutionary history. The resulting gene expression data were used to perform a correlation network analysis to test the extent to which this co-functional network is conserved across plant lineages (Fig. 3D, Supplementary Data Table S14).

Doxycycline can induce mitochondrial stress and activate UPR^mt^ [9, 40, 41]. We treated mature leaves of *A. thaliana*, *O. sativa*, and *S. lycopersicum* with 10 mg ml^−1^ doxycycline to detect the activation of UPR^mt^, which was inferred from the high expression of *MTHSC70-1*/*-2* (Fig. 3E). After 7 days, leaves showed abundant signs of senescence (Fig. 3F), and the expression of leaf senescence-associated genes, such as chlorophyll-degrading genes [non-yellow coloring 1 (*NYC1*), NYC1-like (*NOL*), and red chlorophyll catabolite reductase (*RCCR*)], and others [*ANAC042*, *ANAC083* and senescence-associated gene 13 (*SAG13*)] were significantly upregulated (Fig. 3G). Overall, the results of our analysis indicate that leaf senescence is associated with mitostasis across the major plant lineages and induces systematic UPR^mt^ to mitochondrial stress.

### Transcription factor motif enrichment analysis reveals candidate central regulators of senescence

**Figure 4:**
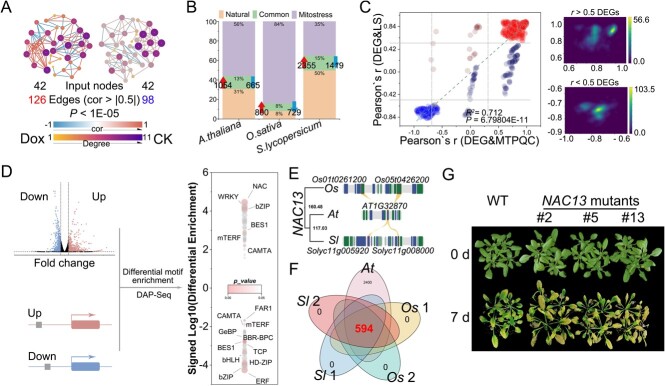
Candidate central regulators of senescence. (A) Correlation networks of the co-functional network of genes and a random network of the same number of genes according to the mitochondrial stress-induced senescence dataset (GSE201607). Network complexity (number of edges) and the correlations (color of edges) were compared (*P* < 1e−05). Nodes correspond to the numbers of input genes. (B) Percentage accumulation of DEGs in the leaves of *S. lycopersicum*, *O. sativa*, and *A. thaliana* under natural senescence or mitochondrial stress-induced senescence. Orange corresponds to DEGs associated with natural senescence, purple indicates DEGs associated with mitochondrial stress-induced senescence, and green corresponds to DEGs associated with both natural senescence and mitochondrial stress-induced senescence. (C) Correlation density map comparing leaf senescence-associated genes and mtPQC according to the senescence dataset (GSE67777). The *y*-axis shows the correlations of DEGs involved in natural leaf senescence and mitochondrial stress-induced leaf senescence with leaf senescence-associated genes; the *x*-axis shows the correlations of DEGs involved in natural leaf senescence and mitochondrial stress-induced leaf senescence with mtPQC-associated genes. Red corresponds to upregulated DEGs and blue to downregulated DEGs. (D) Candidate central regulators of senescence discovered by TF motif enrichment analysis in *S. lycopersicum*, *O. sativa*, and *A. thaliana*. The degree of enrichment is indicated by point size, and the *P*-value is indicated by color depth. (E) Schematic diagram of the phylogenetic relationships of *NAC13* among *S. lycopersicum*, *O. sativa*, and *A. thaliana*. (F) Venn diagram of *NAC13*-related functions in *S. lycopersicum*, *O. sativa*, and *A. thaliana* according to M-MAD. (G) Phenotypes of *NAC13* mutants following dark treatment for 7 days.

We obtained the transcriptomes of *S. lycopersicum*, *O. sativa*, and *A. thaliana* during natural leaf senescence and mitochondrial stress-induced leaf senescence (GSE201607) to clarify the conserved role of UPR^mt^ in leaf senescence. The mitostasis-related genes related to natural leaf senescence were more closely associated with the mitostasis-related genes related to mitochondrial stress-induced leaf senescence compared with those identified in the control, which supports a close link between leaf senescence and mitostasis (Fig. 4A, Supplementary Data Table S15). Differentially expressed genes (DEGs) accounted for only 8–15% of the total number of genes identified according to a comparative analysis of data from various species with natural senescence data (Fig. 4B). However, leaf senescence-associated gene expression was significantly correlated with the expression of genes involved in mtPQC (set of upregulated genes, *r* ~ 0.93; set of downregulated genes, *r* ~ −0.78) (Fig. 4C, Supplementary Data Table S16), indicating that these mitostasis-related genes are also related to leaf senescence.

We analyzed the differential enrichment of TF motifs of mitostasis-related genes involved in leaf senescence in *S. lycopersicum*, *O. sativa*, and *A. thaliana.* We used the PlantRegMap database (http://plantregmap.gao-lab.org/) to identify regulators of the leaf senescence-associated co-functional network genes [42]. Multiple TFs, including *NAC*s, *WRKY*s, *bZIP*s, and *ERF*s, were discovered to have preserved functions in the control of leaf senescence across species, and *NAC13* was the most significantly upregulated and played the most important regulatory role (Fig. 4D, Supplementary Data Table S17). Phylogenetic analysis revealed that *NAC13* TFs have undergone an expansion in *O. sativa* and *S. lycopersicum* (Fig. 4E), and paralogs of these genes are functionally conserved according to M-MAD (Fig. 4F, Supplementary Data Table S18). *NAC13* TFs, as mitochondrial–nuclear retrograde signals, are known to be important UPR^mt^ effectors for maintaining mitostasis [43–45]. We subjected plants to dark treatment for 7 days to induce senescence and found that, compared with wild-type plants, *NAC13* mutants had more pronounced signs of senescence after 7 days of dark treatment (Fig. 4G), suggesting that leaf senescence was accelerated when mitochondrial–nuclear retrograde signals were deficient, which presumably affected the maintenance of mitostasis. In sum, we identified several mitostasis-related genes involved in leaf senescence, and the cross-species conserved TFs likely act to regulate leaf senescence.

### Mitochondrial ornithine production catalyzed by ARGAH1 significantly delays leaf senescence

**Figure 5:**
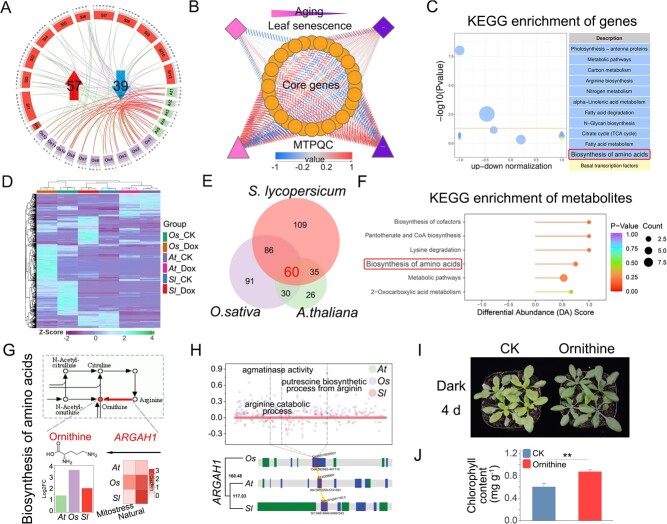
Multi-omics analysis of the conserved biological processes underlying leaf senescence. (A) Cross-species conserved mitostasis-related genes involved in leaf senescence. Green corresponds to *A. thaliana*, purple corresponds to *O. sativa*, and red corresponds to *S. lycopersicum*. A total of 13 downregulated and 19 upregulated single-copy orthogroups were detected. (B) Correlation analysis of cross-species conserved mitostasis-related genes related to mtPQC and leaf senescence according to the senescence dataset (GSE67777) (*P* < .05). The shade of color corresponds to the strength of the correlation. From left to right indicates the aging process. (C) KEGG enrichment analysis of 32 single-copy orthologs. (D) Heat map of the leaf metabolome of *S. lycopersicum*, *O. sativa*, and *A. thaliana* under mitostasis. (E) Venn diagram of distinct sets of metabolites in *S. lycopersicum*, *O. sativa*, and *A. thaliana*. (F) KEGG enrichment analysis of 60 conserved differential metabolites. (G) Genes and metabolites related to amino acid biosynthesis were enriched in the transcriptome and metabolome according to KEGG enrichment analysis. Ornithine and *ARGAH1* were significantly expressed in *S. lycopersicum*, *O. sativa*, and *A. thaliana* during leaf senescence. (H) Schematic diagram of the conserved function of *ARGAH1* in *S. lycopersicum*, *O. sativa*, and *A. thaliana* according to G-MAD. (I) Phenotypes of ornithine-treated plants under 4 days of dark treatment. (J) Chlorophyll content of ornithine-treated plants under 4 days of dark treatment (mg g^−1^). Data are shown as mean ± standard deviation, *n* = 10 (**P < .01).

Among the mitostasis-related genes involved in leaf senescence, we identified 32 single-copy orthogroups across *S. lycopersicum*, *O. sativa*, and *A. thaliana* to clarify the mitostasis-related functions of these conserved genes across species (Fig. 5A, Supplementary Data Table S3), and these genes were indicated as conserved, cross-species mitostasis-related genes involved in leaf senescence. According to the natural senescence dataset (GSE67777), cross-species conserved mitostasis-related genes involved in leaf senescence were closely related to genes involved in mtPQC, which supports a link between the genes involved in leaf senescence and mtPQC (Fig. 5B, Supplementary Data Table S19). Analysis of Kyoto Encyclopedia of Genes and Genomes (KEGG) enrichment of cross-species conserved mitostasis-related genes involved in leaf senescence revealed that their key molecular functions were implicated in metabolic pathways, the tricarboxylic acid cycle, and other pathways (Fig. 5C, Supplementary Data Table S20). These findings prompted us to characterize the metabolomes of *A. thaliana*, *O. sativa*, and *S. lycopersicum* leaves under mitochondrial stress-induced senescence; a total of 328 differential metabolites were identified (Fig. 5D, Supplementary Data Table S21). Analysis of the overlap of the differential metabolites revealed 60 metabolites, including dihydrojasmonic acid, gluconic acid, and hydroxy ricinoleic acid, that were shared among *A. thaliana*, *O. sativa*, and *S. lycopersicum* leaves under mitochondrial stress-induced senescence (Fig. 5E, Supplementary Data Table S21). The molecular functions of these conserved differential metabolites were mainly related to pathways such as biosynthesis of cofactors and lysine degradation according to KEGG analysis (Fig. 5F, Supplementary Data Table S22).

According to the KEGG analysis, the molecular functions of the differential genes and metabolites were primarily enriched in the biosynthesis of amino acids (Fig. 5C and F). Examination of genes related to amino acid biosynthesis revealed ARGININE AMIDOHYDROLASE 1 (*ARGAH1*), which catalyzes the conversion of arginine to ornithine through hydrolysis [46, 47]. Furthermore, ornithine was abundant during leaf senescence in various species (Fig. 5G). G-MAD revealed that functions such as arginine catabolic process of the single-copy orthologs of *ARGAH1* were conserved among *S. lycopersicum*, *O. sativa*, and *A. thaliana* (Fig. 5H, Supplementary Data Table S23). Moderate ornithine supplementation has been shown to delay senescence in animals [48]. When leaf senescence was induced by dark treatment, plants supplemented with 3 mmol l^−1^ ornithine showed delayed leaf senescence after 4 days (Fig. 5I) and had a higher chlorophyll content compared with control plants (Fig. 5J). In sum, ornithine production catalyzed by *ARGAH1* in the mitochondria significantly delays leaf senescence, and this is a conserved function across plant species.

## Discussion

The primary objective of this study was to identify the central regulatory hub underlying the process of leaf senescence across plant species. Using a combination of our own and publicly available data, we found that although it has been observed that leaf senescence tends to evolve gradually in response to various selective pressures [3] and that there is variation in the leaf senescence process among species, there is a high degree of similarity in the pathways and regulators involved in the leaf senescence process. In plants, multicopy orthologs have been generated through both functional divergence and gene duplication events. Functional conservation is observed in only a few orthologs, while others may exhibit functional diversification or EP differentiation. Through phylogenetic and gene expression analysis, we identified orthologs with shared biological roles [49, 50], which resulted in a gene network that could be used to explore the conservation of genes and functions across species [51]. Leaf energy status has been shown to remain stable during leaf senescence [7]. The oxidation–reduction process occurs in the mitochondria and provides energy for cellular processes, and genes related to several EPs were found to be enriched in the oxidation–reduction process. This, coupled with the extensive cellular communication that takes place during senescence, suggests that the mitochondria, organelles that are crucial to cellular signaling and metabolism [52], are also important to the process of leaf senescence.

Genetic, molecular, and biochemical studies of leaf senescence-associates genes are limited, and the resulting data are insufficient to construct a comprehensive gene interaction network. The development of multi-omics approaches has increased the use of network-based approaches for studying complex processes such as leaf senescence [11, 34, 53]. Here, we found that leaf senescence-associated co-functional network genes were enriched in macromolecular material metabolism, abscisic acid response, and UPR^mt^. When macromolecules degrade, nutrients like nitrogen, phosphate, and carbohydrates are released and transferred to organs that are developing and storage tissues [54, 55], and the degradation process appears to be more important than biosynthesis [56]. Abscisic acid is a well-known enhancer of leaf senescence [57]. The unfolded protein response can repair the damage induced by proteotoxic stress, including clearing unfolded protein buildup, in order to maintain cellular homeostasis [9, 40, 58]. Unfolded protein response-associated genes were detected in the co-functional network of genes underlying leaf senescence. When doxycycline was applied to disrupt mitostasis, leaf senescence was accelerated; this indicates a close link between leaf senescence and mitostasis.

Plant UPR^mt^ employ various TFs to regulate mitostasis, such as members of the *AP2*, *WRYK*, and *MYB* families [9]. In plants, the *NAC* TF family is extensive, and *NAC* genes are key regulators of the leaf senescence process. Positive leaf senescence regulators include *NAC092*, *NAC029*, *NAC059*, *NAC081*, *NAC002*, *NAC019*, *NAC055*, *NAC072*, *NAC102*, *NAC032*, *NAC046*, *NAC016*, and *NTL9*; negative regulators of leaf senescence include *NAC042*, *NAC017*, *NAC082*, *NAC083*, *NAC090*, *NTL9*, *NAC082*, *NAC083*, *NAC090*, and *NAC075* [59–75]. We identified several conserved TFs across species, including the mitochondrial–nuclear retrograde signal encoded by *NAC13* [44, 45], which promotes the maintenance of mitostasis and delays senescence. Additional functional studies are needed to explore other putative modulators. Mitochondria play important roles in nitrogen remobilization and release through various catabolic processes [7]. Arginase hydrolysis was significantly enhanced in the mitochondrial urea cycle, which increased the production of ornithine. The metabolic intermediates of the urea cycle are osmoprotective and antioxidant substances that can directly enhance plant stress resistance. They also contribute to various other processes, such as stress response signaling, the regulation of ion homeostasis, and the production of antioxidant and osmoprotective substances as precursors of proline, polyamines, and nitric oxide [76, 77]. Leaf senescence was alleviated following ornithine supplementation; however, the molecular mechanism underlying this process has not yet been elucidated.

### Conclusions

We evaluated the degree of functional conservation in the pathways underlying leaf senescence in a typical horticultural crop (*S. lycopersicum*), a monocot plant (*O. sativa*), and a eudicot plant (*A. thaliana*) via analyses of gene EPs and phylogenetic analyses. A conserved, cross-species leaf senescence-associated co-functional gene network was obtained; a total of 2769 conserved functions were identified. Co-functional gene expression was altered by mitochondrial stress and experimental data indicated that UPR^mt^ underlies the leaf senescence process. TF binding site enrichment analysis revealed the presence of conserved regulators (e.g. NAC, bZIP, and WRKY TFs). Transcriptomic and metabolomic analyses revealed that *NAC13* and ornithine enhance resistance to leaf senescence by promoting the maintenance of mitostasis. Taken together, these results supply new information on the process of leaf senescence and reveal conserved, cross-species regulatory and biological processes (UPR^mt^) which underlie leaf senescence.

## Materials and methods

### Plants and cultivation conditions

The European Arabidopsis Stock Center provided Columbia-0 background *A. thaliana NAC13* (*AT1G32870*) mutants. The quantitative real-time polymerase chain reaction (qRT–PCR) was used to confirm *NAC13* knockdown. *A. thaliana* plants were cultivated on 1/2 Murashige and Skoog medium (pH 5.7) supplemented with 0.75% (w/v) phytoagar and 1% (w/v) sucrose. *Arabidopsis* plants were grown at 22°C under an 8 hours dark/16 hours light photoperiod, at a light intensity of 100 μE m^−2^ s^−1^. Japonica rice (*O. sativa*) cultivar ‘Zhonghua 11’ (ZH11) was grown in a greenhouse under a 12 hours light/12 hours dark photoperiod at 28°C during the light period and 22°C during the dark period. Tomato (*S. lycopersicum*) cultivar AC was grown in pots containing a 1:1:1 peat moss:vermiculite:sand mixture under a 16 hours light/8 hours dark photoperiod at 27°C during the light period and 16°C during the dark period and 68%–75% relative humidity.

### Data sources and sequence retrieval

Publicly available transcriptomic data (Supplementary Data Table S7) were obtained from the National Center for Biotechnology Information. Specifically, transcriptomic data were obtained for several stress conditions, including nutrient stress [78], mitochondrial stress [78], hormone stress [79, 80], chloroplast stress [78], biological stress (*Botrytis cinerea* [79], elicitor Flg22 [79], *Erysiphe orontii* [79], *Phytophthora infestans* [79], *Blumeria graminis f. sp. hordei* [81], elicitor EF-Tu [82], and *Erysiphe cichoracearum* [83]), and abiotic stress (ozone [84], high light [85], H_2_O_2_ [80], heat [79], salt [79], UV [79], oxidative stress [79], and osmotic stress [79]). Furthermore, both CF–MS and DAP-seq data from *A. thaliana* were obtained, as well as 1KP Project transcriptome datasets, *A. thaliana* 1001 Genomes Project (GEO: GSE80744) datasets, and data from 144 natural *A. thaliana* accessions (GEO: GSE43858) (Supplementary Data Table S7).

### Examination of cross-species conserved genes underlying leaf senescence

The STEM program version 1.3.13 [24] was used to analyze EPs using RNA-seq data obtained from the leaves of *O. sativa*, *A. thaliana*, and *S. lycopersicum* during three major periods of leaf development (juvenile, mature, and senescence stages). Leaves from the juvenile period were used as a control. The following criteria were used to identify significantly expressed genes: *P* < .05 and log_2_(FC) > 2. According to the clustering of significantly expressed genes, a total of 3^(*n*−1)^−1 EPs were found to be theoretically possible. We conducted an evolutionary analysis of genes within each EP in *S. lycopersicum*, *O. sativa*, and *A. thaliana*, using OrthoFinder version 2.5.4 [25] with the default settings (e = 10e−3), and quality control procedures were used to evaluate the robustness of the results. Orthologs for the three species were obtained for *S. lycopersicum*, *A. thaliana*, and *O. sativa* within each EP by integrating the gene EPs and phylogenetic relationships. Single-copy orthologs that were expressed exclusively during the senescence stage were considered cross-species conserved genes involved in leaf senescence.

### GeneBridge analysis

The PEER tool was used to preprocess the data in order to eliminate potential confounding variables [86]. Next, the data were transferred to the GeneBridge toolkit for subsequent analysis. Ontology terms, biological pathways, and knowledge-based gene sets from diverse resources are all referred to as ‘modules’ in the GeneBridge toolset; only ontology terms were utilized in this work. For the purpose of speculating on probable gene functions, the GeneBridge tool G-MAD utilizes large-scale cohort expression data. The Correlation-Adjusted MEan RAnk (CAMERA) gene set test, a competitive testing technique used by G-MAD that makes allowances for inter-gene correlations [87], was used to ascertain the connections between relevant genes and biological modules. Depending on the enrichment direction, a binding score of 1 or −1 was given to gene–module interactions exhibiting significant *P*-values after several testing adjustments. Those interactions that did not persist were assigned a binding score of 0. After performing a meta-analysis, the average binding scores were weighted by the within-module inter-genic correlation coefficient ($\overline{p}$) and sample size to provide gene–module association scores.

According to cross-species transcriptome compendia, linkages between modules were found using M-MAD from the GeneBridge toolbox. To examine the relationships between genes and modules, the G-MAD results for each module compared with all genes were used. The gene-level data collected using the CAMERA technique were used to quantify enrichment against all modules using the enrichment scores of all genes for the target modules [87]. The condensed cross-module *P-*values were changed to 1, 0, or −1 based on Bonferroni thresholds, and a meta-analysis was conducted to produce MMASs. Analysis of complete expression datasets was used to build the module association network. By contrast, previously published gene annotations were used to build the module similarity network. Therefore, the module association network permits the discovery of new biological linkages between modules that have no previous documentation.

### Transcriptome analysis

Both RNA-seq and library construction were carried out by Wuhan Metware Biotechnology Co., Ltd (Wuhan, China). The mRNA was purified using a TruSeq RNA Sample Prep Kit (Illumina, USA). Sequencing of the mRNA was conducted using an Illumina HiSeq 6000 sequencer. Using Trimmomatic (v0.39) [88] and FastQC (v0.11.9) [89] to filter and trim raw RNA readings, clean reads were produced. HISAT2 was used to draft high-quality reads to the draft reference genome. FeatureCounts (v1.6.3) [90] was used to quantify gene expression (transcripts per million) (Supplementary Data Table S24). The DESeq2 package [91] was used to normalize gene expression levels (BaseMean), and an adjusted *P*-value (*P*_adj_) < .05 was used as the threshold to identify DEGs for each comparison group.

### Metabolite profiling

A liquid chromatography (LC)-electrospray ionization (ESI)–MS/MS system (high-performance LC, Shim-pack UFLC Shimadzu CBM30A system; MS, Applied Biosystems 4500 QTRAP) was used to analyze sample extracts, according to the following: Waters ACQUITY UPLC HSS T3 C18 (2.1 × 100 mm, 1.8 μm) column; 40°C; 5 μl injection volume; gradient 100:0 v/v at 0 minutes, 5:95 v/v at 10.0 minutes, 5:95 v/v at 11.0 minutes, 95:5 v/v at 11.1 minutes, and 95:5 v/v at 15.0 minutes. The solvent was water (0.04% acetic acid):acetonitrile (0.04% acetic acid), and the flow rate was 0.4 ml/minute. Alternatively, the effluent was connected to an ESI-triple quadrupole-linear ion trap (QTRAP)-MS. Linear ion trap and triple quadrupole scans were obtained on a QTRAP-MS using an API 4500 QTRAP LC/MS/MS system. According to the metabolites eluted within each period, monitoring of a specific set of multiple reaction-monitoring transitions was conducted.

### Gene Ontology enrichment analysis

A GO enrichment analysis of the single-copy orthologs of the EPs was conducted using the goseq R package release 3.15. Statistically significant enrichment was attained at *P*-values <.05. WEGO software was used to functionally classify GO annotations and determine functional distribution.

### qRT–PCR analysis of gene expression

The qRT–PCR reactions were carried out according to the following conditions: predenaturation at 95°C for 3 minutes, followed by 40 cycles of denaturation at 95°C for 5 seconds and annealing and extension at 60°C for 30 seconds. Primers were designed using Primer3 (http://frodo.wi.mit.edu/). Gene expression levels were quantified using the 2^-ΔΔCT^ method. Three biological replicates, each consisting of three technical replicates, were performed.

### Analysis of differential transcription factor binding site enrichment

The FunTBS method, with a threshold *P*-value ≤.05, was used to identify differential TF binding site enrichment using the Plant Transcriptional Regulatory Map database (http://plantregmap.gao-lab.org/tf_enrichment.php). Based on prior research and ChIP-seq data, differently enriched TF binding sites were found or inferred using information on TF binding motifs and regulatory elements. To determine if the fraction of genes targeted by TFs was higher than the background level, Fisher’s exact tests were used.

### Correlation network construction and visualization

The correlation network was created using the R package imsbInfer from GitHub. Correlations were calculated using Pearson’s rank correlation measure, with red edges indicating positive correlations and blue edges indicating negative correlations.

### Statistical analysis

Origin Pro 2021 software (OriginLab Corporation, USA) was used to analyze experimental data. Statistically significant differences between groups were determined using least significant difference tests, with threshold *P*-values of .01 and .05.

## Acknowledgements

This research was sponsored by the National Natural Science Foundation of China (32071160; 32161133021; 32101682), Shanghai Association for Science and Technology (20ZR14279; 20YF1422000), and Shanghai Jiao Tong University (20X100040052). We thank TopEdit (www.topeditsci.com) for linguistic assistance during the preparation of this manuscript.

## Author contributions

M.-Y.L. planned and designed the research. M.-Y.L. and C.-C.G. analyzed the data. M.-Y.L. wrote the original manuscript. K.-X.X. and K.C. determined the expression of genes by qRT–PCR. K.-X.X. and Y.-D.W. measured the chlorophyll content. C.-C.G., J.-H.C., and K.C. used the method to integrate plant data. X.W. reviewed and edited the manuscript. M.-Y.L., J.-H.C., and Y.-D.W. revised the manuscript. X.W. and Y.-D.W. supervised the research. All authors read and approved the final manuscript.

## Data availability

All RNA-seq data were deposited at NCBI under GEO Series GSE201607, Sample accession numbers GSM6068492-GSM6068509, and Experiment/Run accession number SAMN27783488-SAMN27783505.

## Conflict of interest

The authors have no conflict of interest to declare.

## Supplementary data

Supplementary data is available at *Horticulture Research* online.

## Supplementary Material

Web_Material_uhac251
